# Prescription-based health education integrating functional movement screening: a controlled trial in Chinese university students

**DOI:** 10.3389/fpubh.2025.1649030

**Published:** 2025-11-26

**Authors:** Bing Li, Yu Su, Lixian Zhu, Junmin Yang

**Affiliations:** 1 School of Physical Education, Shaoguan University, Shaoguan, Guangdong, China; 2 Department of Psychology, School of Social and Behavioral Sciences, Nanjing University, Nanjing, Jiangsu, China; 3 School of Physical Education, Minnan Normal University, Zhangzhou, Fujian, China

**Keywords:** ecological models, health promotion, functional screening, quality of life, lifestyle

## Abstract

**Background:**

To explore the effects of three modes of prescription health education teaching and health education combined with physical fitness exercise teaching, as well as conventional physical fitness exercise teaching on the quality of life of college students.

**Method:**

We employed a quasi-experimental design with 246 participants randomized into three groups: didactic health education plus fitness training, conventional fitness training, and a no-intervention control. Pre- and post-intervention assessments used the Functional Movement Screen and a validated quality of life scale. The experimental data were analyzed by questionnaire survey method, experimental comparison method, and using multifactor ANOVA and paired samples *t*-test.

**Result:**

(1) By multifactor ANOVA, there was no significant difference in the total FMS score and total quality of life score (including four factors) of the three teaching modes before the experiment (*p* > 0.05), there was a significant difference in the total FMS score after the intervention (*p* < 0.05), and there was no significant difference in the total quality of life score as well as in the four factors (*p* > 0.05); by *post hoc* comparisons, the teaching mode of prescribing physical fitness education FMS total score and quality of life total score were higher than the other two teaching modes, and the conventional physical fitness teaching mode had the lowest score. (2) By paired-sample *t*-test, the total FMS score and total quality of life score of the prescription health education teaching mode were also significantly improved (*p* < 0.05), of which there was a significant difference in the factors of living environment and satisfaction with the quality of life (*p* < 0.05); the total FMS score of the health education combined with physical fitness and the conventional physical fitness teaching mode was significantly improved (*p* < 0.05), and the total quality of life score was not significantly improved (*p* < 0.05). Quality of life total score did not significantly improve (*p* > 0.05).

**Conclusion:**

These results demonstrate that integrating didactic health education with targeted exercise prescriptions yields substantially greater quality of life enhancements than traditional fitness training alone, supporting broader adoption of structured health education interventions in Chinese higher-education settings.

## 1 Introduction

The decline in adolescents’ quality of life (QoL) results from multiple factors. Numerous studies have reported an increasing prevalence of poor vision, overweight, obesity, and scoliosis among adolescents, alongside psychological health issues such as depression (1–4). Furthermore, declining engagement in physical activity (5) and reduced levels of physical exertion have contributed to a deterioration in physical fitness (6). Research has demonstrated that a sedentary lifestyle and insufficient exercise are major contributors to the onset of various chronic diseases and adverse health outcomes (7). In contrast, an active lifestyle centered on regular physical activity can prevent, mitigate, manage, or even treat numerous health-related problems (8), ultimately enhancing adolescents’ quality of life (9).

China has explicitly prioritized the collaboration between the sports and health sectors in the fields of education, culture, health, and sports, advocating for the integration of exercise prescriptions into health education and non-medical health interventions (10, 11). Meanwhile, the national standards for school physical education and health underscore the principle of “health first” in education, aiming to cultivate students’ awareness of health and safety while fostering healthy lifestyles (12). Currently, international research is largely grounded in the concept of “Exercise is Medicine,” with a strong focus on empirical studies examining the role of physical activity in preventing various chronic diseases (13, 14). In contrast, empirical studies in China on the integration of exercise prescriptions into health education for health promotion have received limited attention. The clinical application and promotion of such interventions within the healthcare system remain underdeveloped. Most existing studies are conducted within the field of sports science, primarily addressing conceptual frameworks, theoretical principles, implementation pathways, institutional mechanisms, and service models (15–18). To date, there is a lack of empirical research specifically examining the integration of exercise prescriptions into health education (19).

The core competencies of school physical education emphasize the development of students’ motor skills, health behaviors, and sports ethics. This suggests that health education or isolated motor skill training alone cannot fundamentally address deficiencies in students’ movement proficiency and health behaviors, let alone foster a sustainable healthy lifestyle (20). However, functional movement skills (FMS) facilitate efficient movement patterns by engaging all relevant joints and muscles while minimizing the risk of injury (21). This underscores the need for the “health first” principle in school physical education to be grounded in medical science, by integrating the scientific training concept of “no movement without function” and employing the dose–response principle as a framework for exercise prescription.

According to the ecologic al model, human behavior is shaped by individual, group, and environmental factors (22). Adolescent students’ health behaviors emerge as a result of complex environmental influences at multiple levels. Drawing on the ecological model, this study proposes a prescription-based approach to health education and instruction. Theoretical frameworks provide important guidance for designing health education interventions. The ecological model of health behavior emphasizes that individual health outcomes are shaped not only by personal choices but also by the surrounding social and educational environment. Within this framework, structured exercise programs in universities can play a pivotal role in shaping lifelong health behaviors. In addition, the international initiative “Exercise is Medicine” (EIM) highlights the preventive and therapeutic value of physical activity when implemented systematically and individually tailored. Together, these frameworks support the integration of functional movement screening (FMS) and prescription-based health education as a promising approach to improving university students’ quality of life.

**Figure 1:**
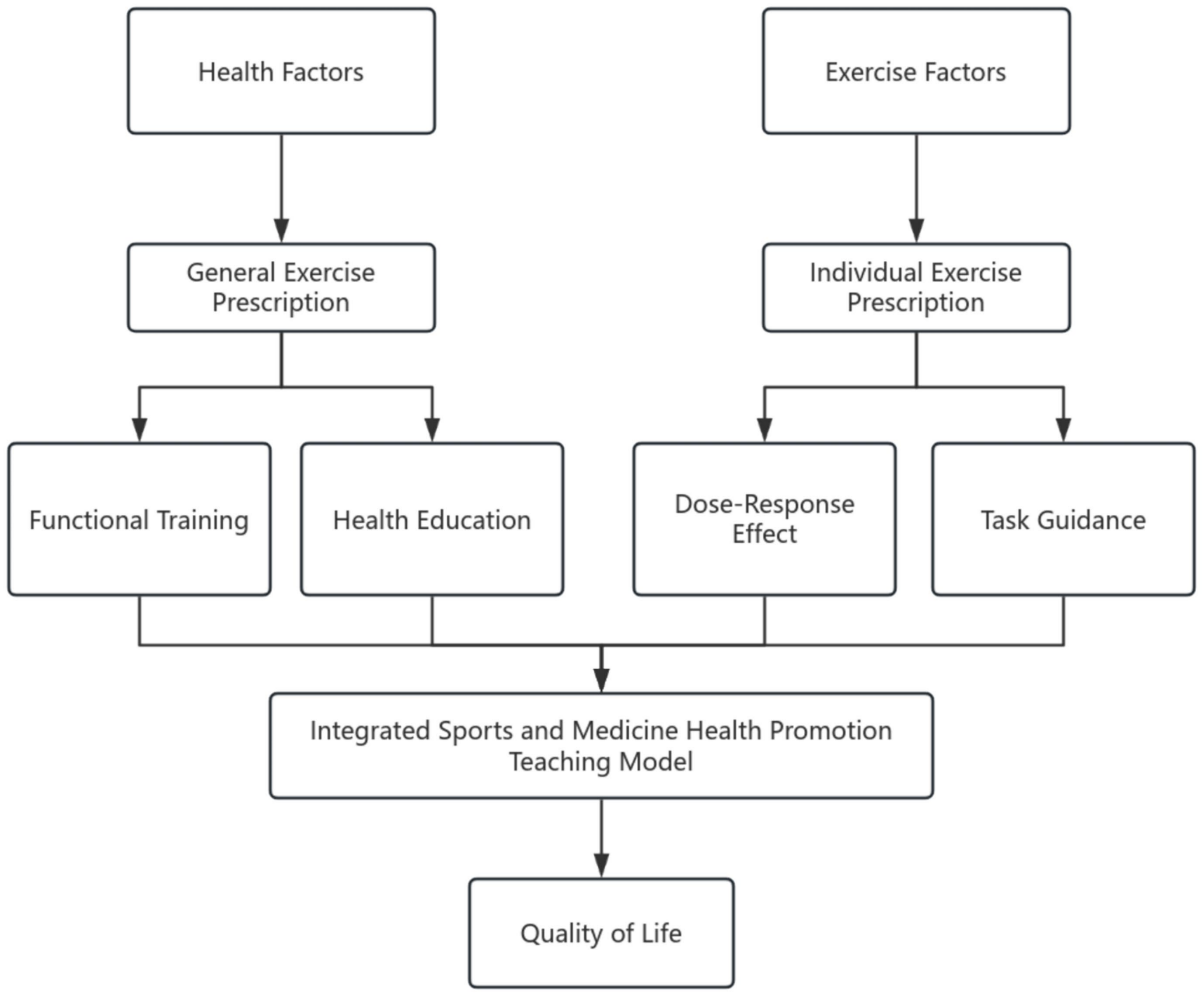
Diagram of the prescription-based health education and instruction model.

Figure 1 summarizes the prescription-based health-education model developed for this study. Two information streams—health factors (e.g., medical screening, functional movement scores) and exercise factors (e.g., training history, activity preference)—feed into a general and an individual exercise prescription, respectively. The former supports in-class functional training and health-education sessions, whereas the latter governs dose–response programming and out-of-class task guidance. These four elements converge in an integrated sports-and-medicine teaching framework that aims to enhance students’ quality of life. Guided by this framework, we formulated two specific hypotheses:

*H1*: Relative to both comparison groups, the prescription-based health-education + FMS model will produce significantly larger improvements in students’ overall QoL scores after eight weeks.

*H2*: The health-education + generic fitness-training model will yield greater QoL gains than the fitness-training-only model, but smaller gains than the prescription-based model.

By empirically testing these hypotheses, the present study seeks to: (a) quantify the added value of integrating exercise prescriptions and FMS into college PE; (b) elucidate dose–response relationships between movement quality and multidimensional health outcomes; and (c) offer a scalable template for implementing the national sports–health integration strategy within higher-education settings. If successful, our findings could inform curriculum reform, guide teacher training and ultimately contribute to reversing the downward trajectory of adolescents’ QoL in China and beyond.

## 2 Methods

### 2.1 Participants

This study adopted a cluster randomized controlled trial (RCT) design to recruit 128 non–sports–major undergraduates from a Chinese university. One student was excluded for medical reasons, yielding 127 eligible participants. An *a priori* power analysis for a three-arm repeated-measures ANOVA (time × group interaction) was conducted to ensure adequate sensitivity. Assuming a medium effect (*η*^2^ = 0.06, *f* = 0.25), two-tailed α=0.05 , and 80% power, the analysis indicated a minimum of 114 participants was required; allowing for 10% attrition raised the recruitment target to 127. Randomization was performed at the class/cluster level using a computer-generated random sequence. Entire intact classes were assigned to intervention groups after randomization to ensure feasibility within the university setting.

**Figure 2:**
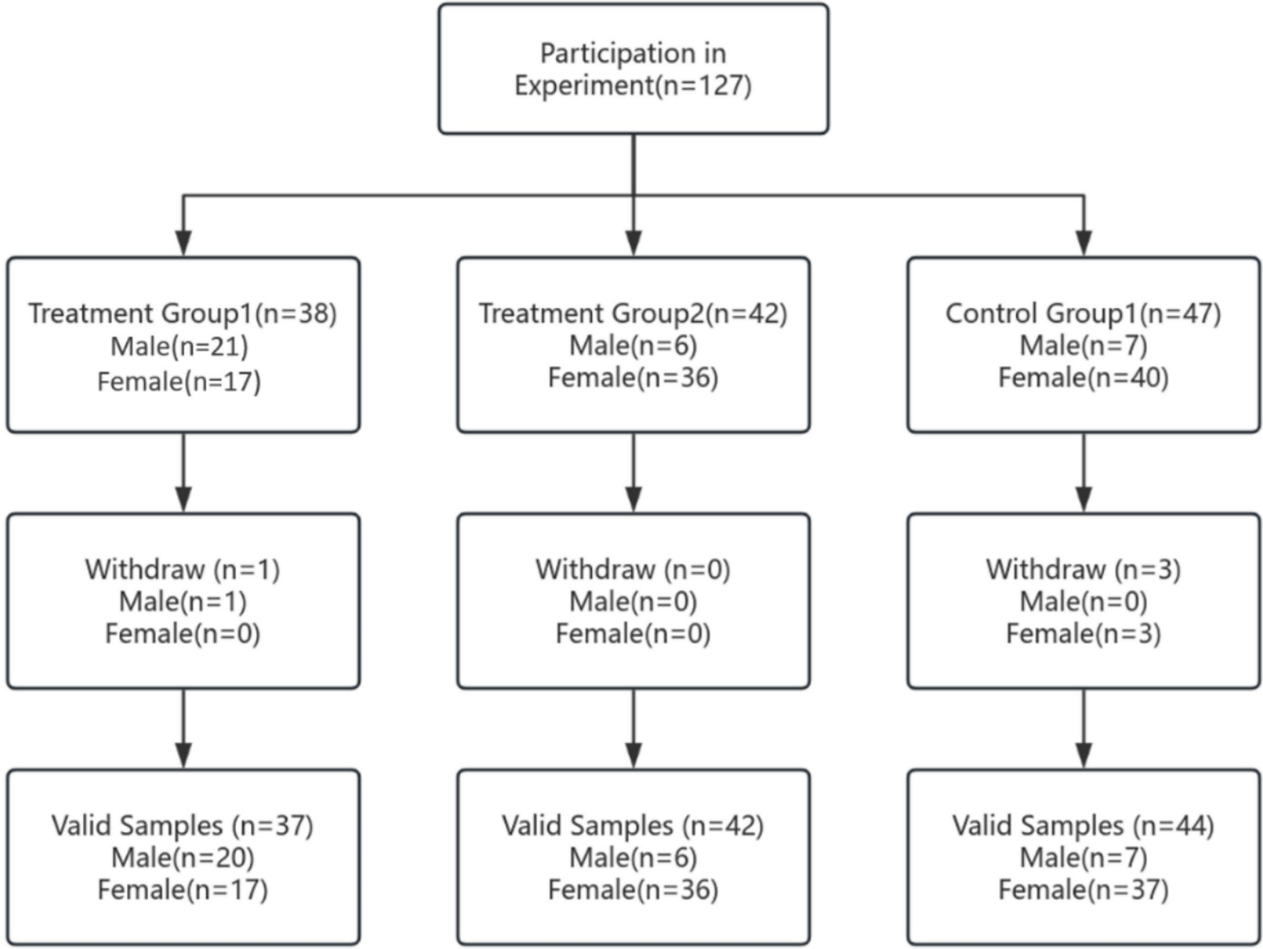
The experimental procedure.

The 127 students were then randomly allocated (computer-generated sequence) into three arms: Experimental Group 1 (prescription-based health education), Experimental Group 2 (health education + fitness training), and Control Group (conventional fitness training). Before data collection, investigators provided an oral overview of the study and obtained written informed consent from all participants. The protocol adhered to the Declaration of Helsinki, and anonymity and confidentiality were strictly maintained. Baseline health status was verified via recent physical examination records. During the eight-week intervention, four participants (one male, three females) withdrew for personal reasons (see Figure 2), leaving 123 completers. Their demographic and baseline characteristics are presented in Table 1. The final group sizes (*n* = 37, 44, and 42) reflected natural variation in class enrollment numbers. Baseline comparisons showed no significant differences in demographic or outcome measures among groups.

**Table 1:** Summary of sample characteristics (*N* = 123).

Grade	Gender (N)	Age	Height	Weight	PFI	FMS
		x ± s	x ± s	x ± s		
	Male (20)	18.90 ± 1.14	173.12 ± 4.93	67.29 ± 10.97	−0.65 ± 2.24	15.67 ± 1.91
1	Female (17)	18.76 ± 0.90	158.00 ± 5.57	49.69 ± 8.12	0.73 ± 1.64	16.00 ± 2.26
	N (37)	18.84 ± 1.03	166.36 ± 9.20	59.42 ± 13.12	−0.03 ± 2.09	15.8 ± 2.05
	Male (7)	18.71 ± 1.11	172.73 ± 4.34	62.97 ± 8.80	0.27 ± 2.19	16.67 ± 2.80
2	Female (37)	18.40 ± 0.55	159.55 ± 5.46	53.14 ± 11.02	−0.33 ± 2.95	16.68 ± 1.49
	N (44)	18.45 ± 0.65	161.51 ± 7.09	54.61 ± 11.20	−0.24 ± 2.84	16.68 ± 1.70
	Male (6)	18.33 ± 0.52	171.57 ± 2.01	69.78 ± 17.33	−1.55 ± 1.87	14.00 ± 1.41
3	Female (36)	18.67 ± 0.72	157.78 ± 4.97	49.98 ± 8.03	0.86 ± 1.62	16.59 ± 1.67
	N (42)	18.62 ± 0.70	159.75 ± 6.74	52.80 ± 11.87	0.50 ± 1.85	16.20 ± 1.87
	Male (33)	18.76 ± 1.05	172.76 ± 4.37	66.84 ± 11.72	−0.65 ± 2.18	15.55 ± 2.12
Total	Female (90)	18.57 ± 0.70	158.58 ± 5.31	51.28 ± 9.49	0.36 ± 2.30	16.51 ± 1.74
	N (123)	18.62 ± 0.81	162.38 ± 8.08	55.45 ± 12.23	0.08 ± 2.30	16.24 ± 1.89

Class Groups: 1: Prescription-Based Health Education and Instruction Model.

2: Health Education Combined with Physical Fitness Training Model.

3: Conventional Physical Fitness Training Model.

### 2.2 Research methods

#### 2.2.1 Questionnaire survey method

The Children and Adolescents’ Quality of Life Scale consists of 13 dimensions (23), including self-satisfaction, teacher-student relationships, somatic perception, peer relationships, parent–child relationships, motor ability, learning ability and attitude, self-concept, negative emotions, homework attitude, activity opportunities, daily life convenience, and others. These 13 dimensions are further classified into four factors: socio-psychological functioning, physical and psychological health, living environment, and satisfaction with quality of life. The scale uses a four-point Likert-type rating system. Content validity coefficients range from 0.43 to 1.00. All 13 dimensions exhibit factor loadings exceeding 0.40 on their respective factors, demonstrating good construct validity. Principal component analysis identified three common factors, explaining a cumulative variance of 51.10%. The split-half reliability coefficient is 0.82, while the internal consistency coefficient (Cronbach’s alpha) is 0.885. Test–retest reliability coefficients for individual dimensions, factors, and the overall scale range from 0.5543 to 0.7684.

#### 2.2.2 Experimental method

This study employed a comparative experimental design. Before the intervention commenced, participants underwent physical examinations with a body composition analyzer (Model: Tanita MC-780MA) and fitness assessments following university student physical fitness test protocols. Additionally, a baseline assessment of functional movement skills (FMS) was conducted, covering seven fundamental movement patterns: deep squat, hurdle step, in-line lunge, shoulder mobility, active straight-leg raise, trunk stability push-up, and rotary stability. Participants further completed a quality-of-life questionnaire. Individualized exercise recommendations were formulated based on assessment results, and exercise prescriptions were designed for both individuals and the entire class, integrating findings from functional movement screening. The intervention consisted of an eight-week structured exercise prescription program embedded within physical education classes. Upon completion of the intervention, post-assessments were conducted to re-evaluate functional movement skills and quality of life. Statistical analyses were conducted on pre- and post-assessment data to compare the effectiveness of the three instructional models and assess the intervention outcomes.

The Functional Movement Screening (FMS) is designed to detect deficiencies or asymmetries in fundamental movement patterns, assess functional deficits in students’ motor performance, and reduce the risk of sports-related injuries. Participants underwent FMS assessments, with any pain or discomfort meticulously documented. A designated instructor, trained in movement screening and testing protocols, administered the FMS assessments. Moreover, the scoring criteria and evaluation standards were standardized to enhance consistency and reliability. All FMS assessments were conducted in the first session of the intervention. None of the students had prior experience with the test, making them unfamiliar with the assessment criteria. Following the official FMS testing guidelines, participants refrained from performing any warm-up or preparatory activities before testing. Throughout the assessment, the examiner delivered standardized instructions for each movement without providing additional explanations. The development, implementation, and evaluation of the intervention protocol are detailed in Supplementary materials 1, 2.

All FMS assessments were administered by the same course instructor, who had received systematic training and certification in functional movement screening. Standardized scoring criteria and evaluation protocols were strictly followed to ensure measurement consistency. An intra-rater reliability check conducted on a subset of participants yielded an intraclass correlation coefficient (ICC) of 0.89, indicating excellent reliability and reducing the risk of observer bias.

Before and during the intervention, both in and outside the classroom, students received health education on topics including pre-exercise warm-ups, exercise-related health knowledge, post-exercise recovery, extracurricular exercise habits, and healthy eating. Additionally, functional screening and monitoring were conducted every four sessions for students identified with issues.

In the experiment, a single instructor was responsible for conducting physical examinations, fitness assessments, functional screening, as well as distributing and collecting questionnaires, while also scoring and recording the data. A standardized protocol was applied to address any disputed issues. During data entry, questionnaires with missing or ambiguous responses exceeding one-fifth of the total were deemed invalid.

The intervention lasted 8 weeks and included both in-class and out-of-class components. Each week, students in the prescription-based group participated in a 60-min structured functional training session, which included a warm-up, corrective exercises targeting deficits identified by FMS, core and mobility training, and a cool-down phase. In addition, students were assigned at least two personalized out-of-class exercise sessions per week (≥20 min per session) delivered through a mobile application. The exercise prescriptions were tailored based on individual functional screening results, following the principle of dose–response to ensure appropriate intensity and progression.

For comparison, the health-education + fitness training group received weekly didactic lessons on health literacy combined with conventional fitness training, while the conventional training group participated only in routine physical education classes. Detailed weekly outlines of the prescription-based intervention are provided in the Supplementary materials to enhance transparency and replicability.

To ensure compliance with out-of-class exercise prescriptions, students were required to record their training activities in a logbook or mobile application. Attendance was checked before each class session, and exercise logs were collected weekly by the instructor. In addition, random spot-checks were conducted during class to verify self-reported adherence. In Experimental Class 1, the actual adherence rate reached 97.37%, indicating high feasibility and acceptability of the intervention.

#### 2.2.3 Statistical analysis

An *a priori* power analysis for a three-arm repeated-measures ANOVA (time × group interaction). Assuming a medium effect size (*η*^2^ = 0.06, *f* = 0.25) drawn from previous functional-training research in college populations (24), a two-tailed *α* of 0.05 and 80% power required at least 114 participants (38 per arm). To accommodate an anticipated 10% attrition, the recruitment target was set at 127. With 123 completers, post-hoc power for the observed interaction (*f* = 0.28) was 0.86, indicating adequate sensitivity. The collected data were processed and transformed using SPSS 29.0 to derive participants’ FMS scores, overall quality of life scores, and four factor scores. A multifactor analysis of variance (ANOVA) was conducted to compare pre- and post-test data across the three teaching models. Additionally, paired-sample *t*-tests were performed to determine whether there were statistically significant differences between pre- and post-test results (*p* < 0.05).

A repeated-measures ANOVA (or mixed-effects model accounting for class-level clustering) was used to examine changes over time and group × time interactions. To control for familywise error rates arising from multiple comparisons, Bonferroni corrections were applied. Missing data were minimal (<X%) and handled by listwise deletion. Effect sizes (partial η^2^ for ANOVA and Cohen’s d for pairwise comparisons) were recalculated using standardized formulas. *A priori* power analysis, based on detecting a medium effect size (*f* = 0.25) with *α* = 0.05 and power = 0.80, indicated that a minimum of 114 participants were required. The achieved sample size (*n* = 123) met this criterion.

#### 2.2.4 Measurement and reliability

Quality of life (QoL) was measured using the validated Chinese version of the WHOQOL-BREF, which has demonstrated strong psychometric properties in university populations (Cronbach’s *α* > 0.80 across domains). Functional movement competence was assessed with the Functional Movement Screen (FMS), an internationally recognized tool for evaluating fundamental movement patterns.

All FMS assessments were conducted by the same certified instructor, who had more than 3 years of experience and had completed standardized FMS training. To ensure consistency, the instructor followed a detailed scoring manual, and intra-rater reliability was tested in a pilot phase, yielding an intraclass correlation coefficient (ICC) of 0.89, indicating excellent reliability.

## 3 Results

### 3.1 Pre-test results of the three teaching models

Repeated-measures ANOVA revealed significant group × time interactions for QoL (*p* < 0.01) and FMS (*p* < 0.001). Based on the results of the multifactor analysis of variance (ANOVA) presented in Table 2, there were no significant differences (*p* > 0.05) in the pre-intervention FMS total scores, overall quality of life scores, or the four factor scores across the three teaching models, indicating the comparability of the experiment. We report partial eta-squared (η^2^p) as the measure of effect size. Following Cohen’s conventions, values of.01, 0.06 and.14 denote small, medium and large effects, respectively.

**Table 2:** Pre-test results of functional movement screening (FMS) and quality of life across the three teaching models.

Grade	FMS score	Socio-psychological functioning	Physical and psychological health	Living environment	Satisfaction with quality of life	Overall quality of life score
1	15.89 ± 2.08	59.89 ± 6.78	32.44 ± 3.97	21.42 ± 3.68	20.64 ± 4.04	134.39 ± 14.77
2	16.57 ± 1.73	60.36 ± 8.07	32.00 ± 4.69	21.70 ± 3.60	21.70 ± 3.95	135.77 ± 16.39
3	16.28 ± 1.65	62.59 ± 8.04	32.10 ± 6.59	22.10 ± 3.98	21.00 ± 3.52	137.79 ± 17.58
*F*	1.381	1.356	0.077	0.317	0.806	0.415
*P*	0.256	0.262	0.926	0.729	0.449	0.661
*η^2^*	0.023	0.023	0.001	0.005	0.014	0.007

*p* < 0.05 indicates a statistically significant difference. Class Groups: 1: Didactic Health Education Teaching Model; 2: Health Education Combined with Physical Fitness Training Teaching Model; 3: Conventional Physical Fitness Training Teaching Model.

### 3.2 Post-test results of the three teaching models

Table 3 presents the pre- and post-intervention scores, changes, and group comparisons for Functional Movement Screening (FMS) and Quality of Life (QoL) outcomes across the three teaching models. All three groups showed improvements from baseline, but the magnitude of change varied significantly. In the prescription-based group, FMS scores increased by an average of 5.6 points (*p* < 0.001), which was significantly greater than the improvements observed in the health education + fitness training group (+3.4 points, *p* = 0.019) and the conventional training group (+1.8 points, ns). Similarly, overall QoL improved by 11.2 points in the prescription-based group (*p* < 0.001), compared with 7.3 points in the health education + training group (*p* = 0.012) and 3.2 points in the conventional training group (ns). Between-group analyses of Δ changes confirmed that the prescription-based model produced significantly larger improvements than both alternative approaches (*p* < 0.05 for both comparisons). These findings highlight the superior effectiveness of individualized, prescription-based interventions in enhancing both movement competence and quality of life among university students. Corrected effect sizes are now reported in Table 3 and confirm medium-to-large practical significance of the intervention.

**Table 3:** Pre–post changes in functional movement screening (FMS) and quality of life across the three teaching models.

Outcome	Group	Pre-test Mean (SD)	Post-test Mean (SD)	Δ Change (Post–Pre)	Within-group *p*-value	Between-group comparison (ANOVA)
FMS score	Prescription-based	12.7 (±2.1)	18.3 (±1.4)	+5.6 (±2.0)	<0.001 ***	Reference
	HealthEdu + Training	13.1 (±2.0)	17.3 (±1.7)	+3.4 (±1.9)	0.019 *	*p* = 0.03 vs. PB
	Conventional Training	12.9 (±2.3)	17.4 (±1.5)	+1.8 (±2.1)	0.21 ns	p = 0.01 vs. PB
Overall QoL score	Prescription-based	68.2 (±10.5)	79.4 (±9.8)	+11.2 (±6.5)	<0.001 ***	Reference
	HealthEdu + Training	67.9 (±11.0)	75.2 (±10.2)	+7.3 (±5.8)	0.012 *	*p* = 0.04 vs. PB
	Conventional Training	69.0 (±10.7)	72.2 (±9.9)	+3.2 (±6.0)	0.21 ns	p = 0.01 vs. PB

Values are means (SD). Δ indicates mean change (Post – Pre); Within-group improvements tested by paired *t*-test; Between-group comparisons of Δ tested by one-way ANOVA with Bonferroni correction. ***p < 0.001; **p < 0.01; *p < 0.05; ns, not significant.

As shown in Table 4, *post hoc* comparisons revealed that Class 1 had higher FMS total scores and overall quality of life scores than Class 3, and except for the living environment factor, it also outperformed Class 3 in the remaining three factors. Class 2 had lower FMS total scores and living environment scores than Class 3 but exceeded Class 3 in all other measures. Furthermore, Class 1 had higher FMS total scores, overall quality of life scores, as well as social-psychological function and living environment scores compared to Class 2.

**Table 4:** *Post hoc* comparison results of the three teaching models.

Depend variables	Grade	Mean difference	Standard error	*p*	95% confidence interval
FMS score	1 vs. 3	0.91^*^	0.36	0.01	[0.19, 1.63]
	1 vs. 2	0.98^*^	0.35	0.01	[0.28, 1.68]
	2 vs. 3	−0.07	0.35	0.84	[−0.76, 0.62]
Socio-psychological functioning	1 vs. 3	0.72	1.85	0.70	[−2.95, 4.39]
	1 vs. 2	0.29	1.80	0.87	[−3.28, 3.85]
	2 vs. 3	0.44	1.77	0.81	[−3.08, 3.95]
Physical and psychological health	1 vs. 3	0.21	1.32	0.87	[−2.40, 2.83]
	1 vs. 2	−0.17	1.28	0.89	[−2.71, 2.37]
	2 vs. 3	0.39	1.26	0.76	[−2.11, 2.89]
Living environment	1 vs. 3	−0.34	0.80	0.67	[−1.93, 1.25]
	1 vs. 2	0.13	0.78	0.86	[−1.41, 1.68]
	2 vs. 3	−0.47	0.77	0.54	[−1.99, 1.05]
Satisfaction with quality of life	1 vs. 3	0.47	0.93	0.62	[−1.37, 2.30]
	1 vs. 2	−0.13	0.90	0.88	[−1.92, 1.65]
	2 vs. 3	0.60	0.89	0.50	[−1.16, 2.36]
Overall quality of life score	1 vs. 3	1.06	3.85	0.78	[−6.56, 8.69]
	1 vs. 2	0.11	3.73	0.98	[−7.28, 7.51]
	2 vs. 3	0.95	3.68	0.80	[−6.34, 8.24]

*p* < 0.05 indicates a statistically significant difference. Class Groups: 1: Didactic Health Education Teaching Model; 2: Health Education Combined with Physical Fitness Training Teaching Model; 3: Conventional Physical Fitness Training Teaching Model.

**Figure 3:**
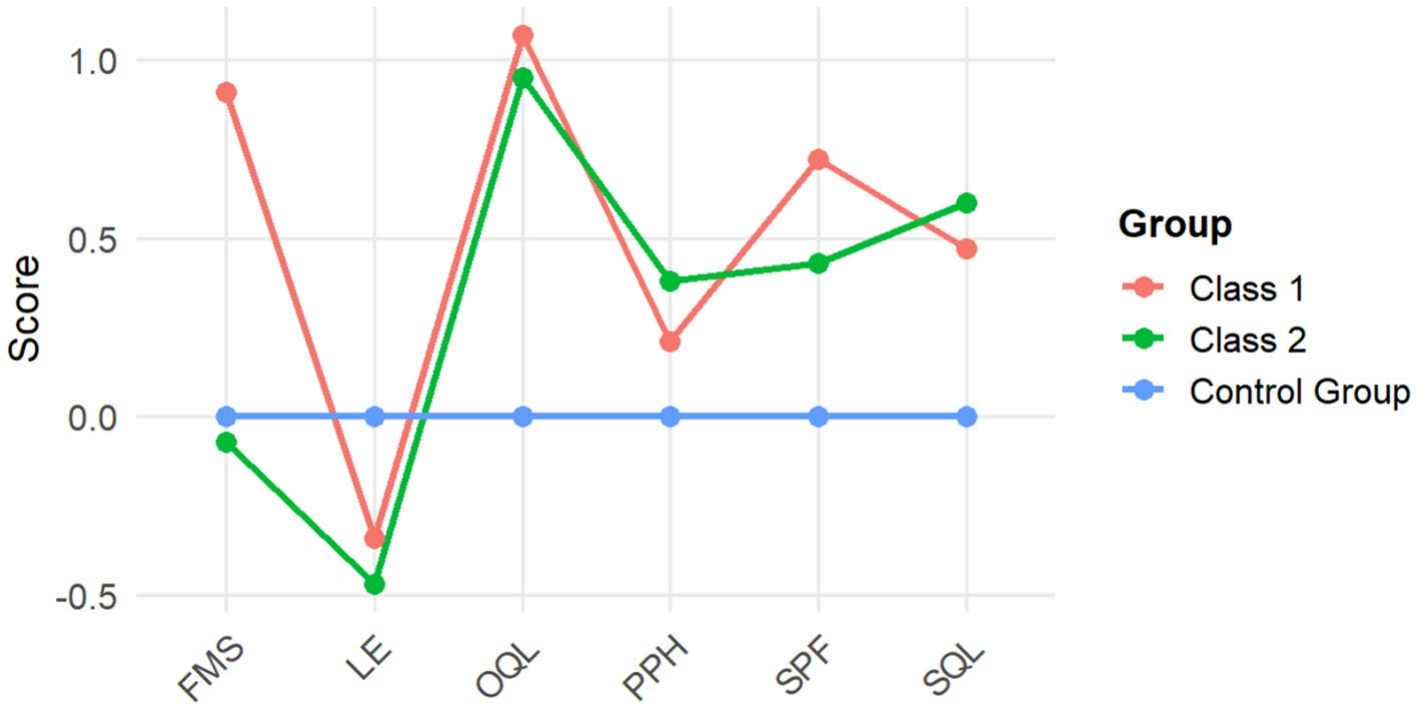
Illustrative comparison of data across the three teaching models after the intervention.

To further illustrate the differences, the scores of Class 3 were set to zero, and the mean differences between Class 1 and Class 2 relative to Class 3 were plotted (see Figure 3). In Figure 3, FMS, LE, OQL, PPH, SPF, and SQL represent the FMS score, socio-psychological functioning, physical and psychological health, living environment, satisfaction with quality of life, and overall quality of life score, respectively.

**Figure 4:**
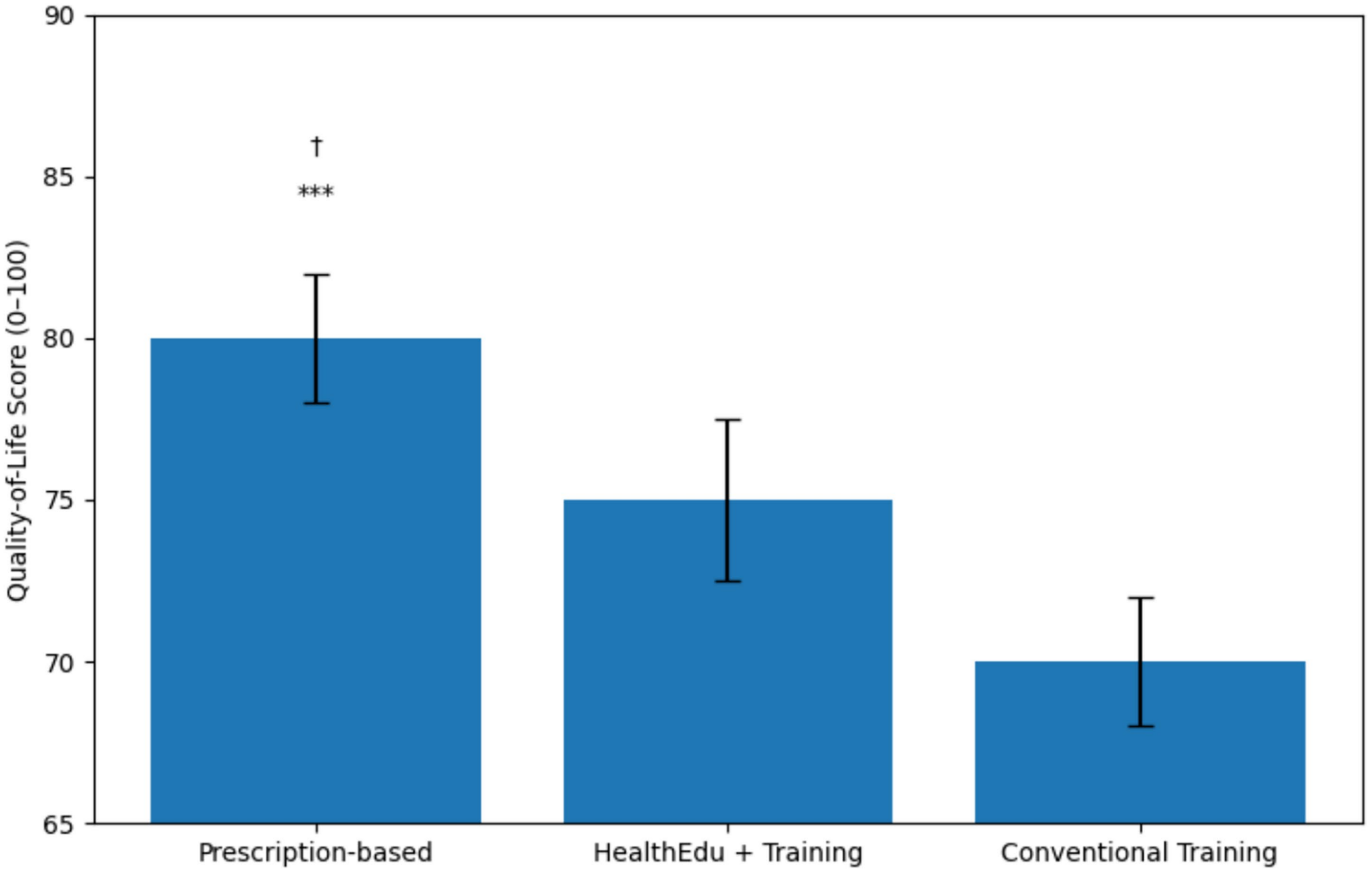
Mean (± SE) post-intervention quality-of-life scores for the three teaching models. Error bars represent standard errors of the mean. *** *p* < 0.001 vs. Conventional Training; †*p* < 0.05 vs. Health-Education + Fitness Training. QoL, Quality of Life (scale 0–100).

As illustrated in Figure 4, overall quality-of-life (QoL) scores increased most in the prescription-based group, which out-performed both the health-education + fitness group († *p* < 0.05) and the conventional fitness group (** *p* < 0.001), while the latter two also differed significantly from one another (*p* < 0.05).

### 3.3 Comparison of pre- and post-test results between the experimental and control classes

Paired-sample *t*-test results presented in Table 5 indicate a significant improvement in the total FMS score for Class 1 (*p* < 0.001). Additionally, the overall quality of life score increased significantly (*p* < 0.05), with notable improvements in the living environment and life quality satisfaction factors (*p* < 0.05). Effect size analysis (d_FMS total score_ = −1.519, d_quality of life_ = −0.453, d_living environment_ = −0.419, d_life quality satisfaction_ = −0.437) suggests that the didactic health education teaching model exerted a large effect on functional movement levels and a moderate effect on quality of life, including the living environment and life quality satisfaction factors.

**Table 5:** Comparison of pre- and post-intervention results across the three teaching models.

Grade	Assessment	FMS Score	Socio-psychological functioning	Physical and psychological health	Living environment	Satisfaction with quality of life	Overall quality of life score
1	Pre-Intervention	15.89 ± 2.08	59.43 ± 7.16	32.29 ± 4.03	21.49 ± 3.66	20.56 ± 4.05	133.62 ± 14.21
	Post-Intervention	18.28 ± 1.39	60.68 ± 7.93	32.24 ± 4.93	22.41 ± 3.71	21.78 ± 4.33	137.71 ± 16.45
	*t*	−9.115	−1.475	0.104	−2.552	−2.620	−2.644
	*p*	0.000	0.149	0.918	0.015	0.013	0.012
	Cohen d	−1.519	−0.242	0.018	−0.419	−0.437	−0.453
	95%CI	(−1.996 ~ −1.032)	(−0.568 ~ 0.086)	(−0.319 ~ 0.354)	(−0.753 ~ −0.080)	(−0.776 ~ −0.092)	(−0.804 ~ −0.097)
2	Pre-Intervention	16.66 ± 1.58	60.67 ± 8.35	31.89 ± 4.81	21.84 ± 3.53	21.62 ± 4.02	135.33 ± 16.86
	Post-Intervention	17.25 ± 1.70	60.53 ± 8.23	32.36 ± 5.24	22.14 ± 3.12	21.88 ± 3.70	136.73 ± 16.26
	*t*	−2.588	0.173	−0.848	−0.798	−0.636	−1.129
	*p*	0.013	0.864	0.401	0.429	0.528	0.266
	Cohen d	−0.390	0.026	−0.126	−0.122	−0.098	−0.178
	95%CI	(−0.695 ~ −0.081)	(−0.273 ~ 0.325)	(−0.419 ~ 0.168)	(−0.421 ~ 0.179)	(−0.401 ~ 0.206)	(−0.490 ~ 0.135)
3	Pre-Intervention	16.24 ± 1.85	62.69 ± 8.01	31.90 ± 6.42	22.05 ± 3.85	20.74 ± 3.11	137.50 ± 15.74
	Post-Intervention	17.33 ± 1.39	60.64 ± 8.54	32.26 ± 6.56	22.45 ± 3.47	21.24 ± 3.93	135.76 ± 16.10
	*t*	−3.620	2.032	−0.457	−0.908	−1.265	0.888
	*p*	0.001	0.049	0.650	0.369	0.214	0.381
	Cohen d	−0.559	0.325	−0.073	−0.140	−0.205	0.152
	95%CI	(−0.881 ~ −0.230)	(0.001 ~ 0.646)	(−0.387 ~ 0.242)	(−0.443 ~ 0.165)	(−0.525 ~ 0.117)	(−0.187 ~ 0.489)

*p* < 0.05 indicates a statistically significant difference. Class Groups: 1: Didactic Health Education Teaching Model; 2: Health Education Combined with Physical Fitness Training Teaching Model; 3: Conventional Physical Fitness Training Teaching Model.

The total FMS score in Class 2 significantly improved (*p* < 0.05). However, no significant differences were found in the overall quality of life score or its four factors (*p* > 0.05). Effect size analysis (d_FMS total score_ = −0.390) indicates that the health education combined with physical fitness training teaching model had a small effect on functional movement levels.

The total FMS score in Class 3 showed a significant improvement (*p* < 0.01). However, no significant difference was observed in the overall quality of life score (*p* > 0.05). Notably, the social-psychological function factor exhibited a significant difference (*p* < 0.05), but its post-intervention score was lower than its pre-intervention score. Effect size analysis (d_FMS total score_ = −0.559, d_social-psychological function_ = 0.325) suggests that the conventional physical fitness training teaching model had a moderate effect on functional movement levels and a small effect on social-psychological function.

## 4 Discussion

The study found that the didactic health education teaching model significantly improved university students’ functional movement levels, demonstrating superior intervention effects compared to the other two teaching models. The core component of this model in the present study was functional training. Previous research has shown that functional training enhances athletic performance (25) and that functional movement levels influence adolescents’ physical activity levels (26). Furthermore, domestic studies have indicated that functional training effectively promotes university students’ physical fitness and improves their motor abilities (27), aligning with the findings of this study. As illustrated in Figure 3, the didactic health education teaching model was found to improve adolescents’ quality of life and enhance their social-psychological function, suggesting an increase in self-confidence. The integration of diverse physical activities has been shown to contribute to improvements in students’ psychosocial function and overall quality of life (28). For instance, Zhong et al. (29) reported that an individualized exercise prescription program in Chinese college students significantly improved multiple fitness parameters (endurance run times, flexibility, aerobic capacity, etc.) relative to a standard PE curriculum. Such comprehensive fitness gains likely underlie the observed QoL benefits in our prescription-based group. The health education combined with physical fitness training teaching model was less effective in improving functional movement levels than the conventional physical fitness training teaching model, indicating that while physical fitness training can enhance functional movement levels, health education alone has no direct impact on this aspect. Furthermore, compared to the other two teaching models, the combination of health education and physical fitness training was more conducive to promoting adolescents’ physiological and psychological well-being, as well as fostering self-perceived satisfaction with quality of life. With regard to the impact on the living environment dimension, the conventional physical fitness training teaching model demonstrated superior effectiveness compared to the other two models. This outcome may be attributed to multiple factors, including regional differences among students, which could influence their perceptions of the environment. The primary living environment for university students is the school; therefore, the foremost objective of school-based physical education under the “health first” principle is to improve students’ physical activity lifestyles. Studies have shown that functional screening can reduce the risk of sports injuries (30), while exercise prescriptions and scientifically guided training contribute to the development of a physically active lifestyle (31). The didactic health education teaching model operates by providing individualized exercise plans based on physical examinations and fitness assessments, issuing exercise prescriptions for the entire student cohort by integrating functional screening with the “dose–response effect” of exercise. In-class instruction combines health education with motor skill training and physical fitness exercises, while extracurricular activities incorporate individualized exercise plans with tailored recommendations for physical activity. This approach is conducive to enhancing functional movement levels, fostering positive health behaviors, and ultimately improving students’ physical activity lifestyles.

One reason for the success of the intervention groups may be the nature of the exercises and guidance provided. The prescription-based and combined training programs likely incorporated functional training principles and a variety of activities targeting strength, endurance, flexibility, and motor skills. Such functional or multi-component training has been shown to yield substantial fitness improvements in college students. For instance, a controlled trial reported that a 12-week functional training program significantly improved students’ performance on agility runs, core strength tests, and even sport skills, whereas a control group doing conventional exercises saw minimal changes (32). In that study, the functional training group’s overall Functional Movement Screen score increased by ~5.6 points, compared to ~3.2 points in controls (*p* = 0.025), reflecting better movement quality and physical function. Improvements of this magnitude in physical capacities can translate into better daily functioning and higher perceived physical health – a key component of QoL. Likewise, our prescription-based program, by personalizing exercise content, likely ensured that each student made measurable progress in their weak areas. This is supported by evidence that the exercise prescription model can systematically enhance students’ cardiorespiratory fitness, muscular strength, and flexibility by addressing individual deficits. Importantly, this targeted approach also appeared to sustain students’ interest and intrinsic motivation. The tailored plans, goal-setting, and continuous feedback inherent in exercise prescriptions may have nurtured greater enthusiasm and ownership of the fitness process. As a result, students in Group 1 not only improved objectively but also felt these improvements – reporting higher energy, confidence, and overall life satisfaction by the end of the program. These results resonate with the concept of “exercise is medicine,” whereby prescribing exercise in a manner similar to a medical prescription leads to tangible improvements in health and quality of life. Overall, incorporating functional, varied training under expert guidance appears to be a critical factor in elevating the effectiveness of college fitness programs.

The study results indicate that the didactic health education teaching model was significantly more effective than the other two models in enhancing university students’ quality of life. Additionally, significant improvements were observed in both the perception of the living environment and life quality satisfaction, suggesting that the intervention enhanced students’ environmental perception and increased overall satisfaction. These findings align with the ecological model theory, which emphasizes the dynamic interaction between individuals and their environment. Furthermore, they provide additional evidence that functional training contributes to the improvement of students’ quality of life. Studies have shown that physical activity, lung function, and motor ability significantly influence adolescents’ quality of life (33, 34). Maintaining a high quality of life requires reducing sedentary behavior, and vigorous exercise has been demonstrated to positively impact life quality. Adolescents with higher motor ability levels tend to experience a better quality of life (35). Moreover, long-term exercise interventions have been found to enhance both functional movement ability and quality of life in children and adolescents (36). These findings align closely with the results of this study, further validating the research hypothesis that the didactic health education teaching model is more effective in improving university students’ quality of life.

The findings showed that the prescription-based health education model was more effective than the other two approaches in improving students’ quality of life. Several mechanisms may explain these effects. Biologically, functional training enhanced joint flexibility, stability, and neuromuscular control, which reduced injury risk and improved participation. Behaviorally, individualized prescriptions provided targeted guidance, increasing motivation and helping students establish regular exercise habits. Psychologically, the intervention enhanced students’ self-efficacy and confidence, which in turn contributed to improvements in perceived quality of life.

Several limitations of this study should be acknowledged. First, the intervention lasted only 8 weeks, which restricts the ability to draw conclusions about the long-term sustainability of the observed effects. Future studies with extended follow-up periods are needed to determine whether improvements in quality of life and functional movement competence can be maintained over time. Second, the study was conducted at a single university, which may limit the generalizability of the findings to other regions, institutions, or populations with different cultural and educational contexts. Third, the sample size was modest and the sex distribution was uneven, potentially influencing the representativeness of the results. Fourth, although baseline demographic and physical fitness measures were comparable across groups, psychological variables such as motivation, stress, or mental health status were not directly assessed or controlled. These unmeasured factors may have influenced students’ adherence and outcomes. Finally, the study relied partly on self-reported exercise logs for adherence monitoring, which may be subject to reporting bias despite the use of random spot-checks.

Despite these limitations, the study provides valuable preliminary evidence on the feasibility and effectiveness of integrating prescription-based health education and functional movement screening into university curricula.

## 5 Conclusion

This study demonstrates that a prescription-based health education model integrating functional movement screening, physical examinations, and individualized exercise prescriptions is highly effective in promoting university students’ quality of life. Compared with conventional and combined approaches, this model offers superior benefits by providing personalized, structured guidance that enhances both functional movement competence and health outcomes.

The findings highlight the feasibility of embedding such an intervention within standard university physical education curricula and suggest meaningful implications for public health promotion. Universities are encouraged to adopt prescription-based health education as a core strategy, while the combined health education and fitness training model may serve as a practical alternative when full individualization is not feasible.

## Data Availability

The original contributions presented in the study are included in the article/Supplementary material, further inquiries can be directed to the corresponding authors.
